# Monocrotaline-mediated autophagy via inhibiting PI3K/AKT/mTOR pathway induces apoptosis in rat hepatocytes

**DOI:** 10.3389/fphar.2024.1499116

**Published:** 2024-10-18

**Authors:** Yazhou Guo, Yang Yuan, Ruibo Wang, Jun Bai, Yanqing Jia, Xinxin Qiu, Huafeng Niu, Long Li, Yan Luo, Baoyu Zhao, Zhencang Zhang

**Affiliations:** ^1^ Shaanxi Engineering Research Center of the Prevention and Control for Animal Disease, Yangling Vocational and Technical College, Yangling, Shaanxi, China; ^2^ Shaanxi Engineering Research Center for Forest Musk Deer Industry, Yangling Vocational and Technical College, Yangling, Shaanxi, China; ^3^ The Youth Innovation Team of Shaanxi Universities, Yangling Vocational and Technical College, Yangling, Shaanxi, China; ^4^ Key Laboratory for Efficient Ruminant Breeding Technology of Higher Education Institutions in Shaanxi Province, Yangling Vocational and Technical College, Yangling, Shaanxi, China; ^5^ College of Veterinary Medicine, Northwest A&F University, Yangling, Shaanxi, China

**Keywords:** monocrotaline, hepatotoxicity, autophagy, PI3K/Akt/mTOR signaling pathway, apoptosis

## Abstract

Monocrotaline (MCT), a major pyrrolizidine alkaloid, is well-known for its high liver toxicity. Dysregulation of autophagy induced apoptosis can lead to various liver diseases, including those induced by chemical compounds. Therefore, we aim to explore whether autophagy might serve as a potential strategy for addressing liver apoptosis caused by MCT. In primary rat hepatocytes (PRHs), MCT significantly increased the number of autophagosomes and the expression levels of LC3II, Becline-1, and Atg5, while it decreased the expression of p62 in a concentration-dependent manner at doses of 100, 200, 300, and 400 μM. Western blot assays revealed MCT inhibited the phosphorylation levels of the PI3K/AKT/mTOR pathway. To elucidate the role of autophagy in mediating MCT-induced apoptosis, we further pretreated PRHs with the autophagy agonist Rapamycin and the inhibitors Bafilomycin A1 and Chloroquine, respectively, and assessed the apoptosis of PRHs induced by MCT. The results displayed that Rapamycin increased the apoptosis rate and the expression of cleaved caspase-3, whereas Bafilomycin A1 and Chloroquine reduced the apoptosis and the expression of cleaved caspase-3 in PRHs. This study confirms that autophagy enhances PRHs apoptosis induced by MCT. In summary, this study demonstrates that MCT-induced autophagy via inhibition of the PI3K/AKT/mTOR pathway can lead to apoptosis in PRHs.

## 1 Introduction

Pyrrolizidine alkaloids (PAs) are a group of natural compounds found in approximately 3% of flowering plants (Schrenk et al., 2020). It is crucial to emphasize that more than 50% of these PAs have been proven to possess hepatotoxic effects, leading to their classification as hepatotoxic PAs (HPAs) (Prakash et al., 1999). In addition, some PAs also exhibit nephrotoxicity, teratogenicity, carcinogenicity, and genetic toxicity, with a few even exhibiting pulmonary toxicity. Currently, it has been reported that more than 6,000 species of plants from 13 genera in natural grasslands contain PAs (Smith and Culvenor, 1981; Stegelmeier et al., 1999). These plants are predominantly found in toxic plant families such as *Compositae*, *Boraginaceae*, and *Leguminosae* (Dusemund et al., 2018). Humans are often poisoned by consuming grains or animal-derived foods contaminated with PAs, such as honey, milk, and eggs (Huybrechts and Callebaut, 2015; Mulder et al., 2018; Roncada et al., 2023). Additionally, poisoning can occur from drinking tea or herbal preparations containing PAs. Livestock are forced to consume plants containing PAs during seasons when edible forage is severely scarce, leading to chronic poisoning, and even death, which poses a significant threat to the economic sustainability of grassland livestock farming (Guo et al., 2020).

Monocrotaline (MCT) belongs to the PAs and is mainly derived from plants in the *Crotalaria* genus (Roeder, 1995). Its structure is a macrocyclic diester consisting of 11 members and lacking an α, β-unsaturated double bond. MCT exhibits low toxicity, but after metabolism in the liver, it forms intermediate metabolites that can rapidly bind to nucleophilic substances, such as proteins contenting thiol (-SH), hydroxyl (-OH), and amino (-NH) groups, as well as DNA and RNA, leading to liver damage (Gong et al., 2023). However, the mechanisms of MCT-induced liver injury remain controversial. Our previous study have demonstrated that endoplasmic reticulum stress is a key factor contributing to MCT-induced apoptosis in hepatocytes (Guo et al., 2021). Additionally, research has shown that Kupffer cells play a crucial role in MCT-induced liver injury by producing TNF-α (Cao et al., 2022). Therefore, further investigation is required to fully understand the mechanisms underlying MCT-induced hepatocyte damage.

MCT can induce hepatocyte death (Copple et al., 2004). Cell death can occur through programmed cell death (PCD), including mainly apoptosis, autophagy, necroptosis and ferroptosis. Apoptosis is characterized by DNA fragmentation, chromatin condensation, membrane blebbing and cell shrinkage. During apoptosis, proteins encoded by the Bcl-2 gene family, such as Bcl-2, Bax, along with caspases like caspase-3 and caspase-9, serve as crucial mediators and key executors of the process. Previous studies have found that MCT-induced endoplasmic reticulum stress is one of the causes of hepatocyte apoptosis (Guo et al., 2021). Autophagy, another form of PCD, utilizes autophagosomes to break down unnecessary or damaged organelles, as well as cells exposed to toxins. This process is dynamically regulated by several proteins that coordinate the formation of the autophagic membrane, the engulfment of autophagosomes, and their subsequent fusion with lysosomes, involving key players such as LC3 and p62 (Gao et al., 2020; Allaire et al., 2019; Ichimiya et al., 2020). Nervosine VII, a compound belonging to PAs and extracted from *Liparis nervosa*, has been found to induce autophagy in human colorectal cancer cells (Huang et al., 2020). Nevertheless, the mechanism by which MCT triggers autophagy in the liver remains elusive. In this paper, we observed the effect of MCT on autophagy by examining the PI3K/AKT/mTOR pathway. Additionally, by introducing autophagy inhibitors and agonists, we further investigated the impact of MCT on cell apoptosis, thereby investigating the role of autophagy in MCT-induced hepatocyte apoptosis.

## 2 Materials and methods

### 2.1 Reagent

Monocrotaline (purity >98%, CAS No. 315-22-0) was purchased from Sigma Aldrich (United States, Catalog No. C2401) and the stock concentration of 50 mM MCT was prepared by dissolving in 1 mol/L HCl and balanced the pH to 7.0–7.4 by adding 5 mmol NaOH. DMEM medium (Gibco, United States, Catalog No. 12800017) containing 10% FBS (Zeta Life, United States, Catalog No. Z7181FBS) was used for cell culture. Bafilomycin A1 A1 (Catalog No. HY-100558), Rapamycin (Catalog No. HY-10219) and Chloroquine (Catalog No. HY-17589A) were obtained from MCE, United States. Ad-GFP-LC3B was purchased from Beyotime (Shanghai, China).

### 2.2 Cell culture and drug treatment

Primary rat hepatocytes (PRH) were prepared as previously described (Guo et al., 2021) and male Sprague–Dawley rat was obtained from Cheng Du Dossy Biological Technology Co., Ltd. (Sichuan, China). PRH were seeded at a density of 1 × 10^6^ cells/mL in 6-well plates and maintained in a 37°C incubator with 5% CO_2_. After 24 h cultured, PRH were treated with different concentrations of MCT (0, 100, 200, 300 and 400 μM) for different time (0, 6, 12, 24 and 36 h), or pretreat the cells with Rapamycin (50 nM), Chloroquine (15 μM), or Bafilomycin A1 (10 nM) for 4 h. After removing the medium, replace it with fresh medium containing Rapamycin (50 nM), Chloroquine (15 μM), Bafilomycin A1 (10 nM), Rapamycin + MCT, Chloroquine + MCT, or Bafilomycin A1 + MCT. In the control group, add an equal volume of fresh medium. Incubate the cells at 37°C for an additional 36 h. Then, the cells were collected for subsequent biological analysis.

### 2.3 Western blot analysis

Hepatocytes underwent lysis in an ice-cooled radioimmunoprecipitation (RIPA) buffer fortified with PMSF (R0010, Solarbio, China). Subsequently, the lysates were subjected to centrifugation at 4°C and 12,000 g for 10 min to isolate the supernatant. The protein content within the supernatant was quantified utilizing the BCA assay (PC0020; Solarbio, China). Each sample was then fractionated by SDS-PAGE (10%–15% gel) at 100 V for 1.5 h, followed by transfer onto a polyvinylidene difluoride (PVDF) membrane (Catalog No. BSP0161, PALL, United States). Prior to antibody probing, the membranes were blocked with 5% non-fat milk dissolved in TBS-T for 2 h at ambient temperature. Subsequently, the membranes were incubated overnight at 4°C with the primary antibodies of interest, diluted in an appropriate solution. LC3B (1:1,000, Catalog No.: ab192890), Becline-1 (1:1,000, Catalog No. ab302670), cleaved capase-3 (1:1,000, Catalog No. ab214430) and Atg5 (1:1,000, Catalog No. ab108327) were purchased from Abcam (United States). p62 (1:1,000, Catalog No. 39749), PI3K (1:1,000, Catalog No. 4249), p-PI3K (1:1,000, Catalog No. 17366), AKT (1:1,000, Catalog No. 9272), p-AKT (1:1,000, Catalog No. 4060), mTOR (1:1,000, Catalog No. 2972) p-mTOR (1:1,000, Catalog No. 2971) and β-actin (1:2000, Catalog No. 4970). The membranes were incubated with a goat anti-rabbit IgG-HRP secondary antibody (1:5,000, Beyotime Institute of Biotechnology, China).

### 2.4 Transmission electron microscopy (TEM)

PRH were seeded in 6-well plates and incubated at 37°C for 24 h. The control group was cultured under standard conditions, while the treatment group received 300 μM MCT. After 36 h, cells were harvested, washed three times with PBS, and fixed in 4% glutaraldehyde. Postfixation was carried out in 1% OsO₄ in 0.1 M cacodylate buffer containing 0.1% CaCl₂ for 2 h at 4°C. Samples were stained with 1% Millipore-filtered uranyl acetate, dehydrated with a graded ethanol series, infiltrated, and embedded. Following resin polymerization at 60°C for 48 h, ultrathin sections were cut using an ultramicrotome, stained with 4% uranyl acetate and lead citrate, and imaged with a transmission electron microscope (FEI Tecnai G2 Spirit Bio TWIN, United States).

### 2.5 Fluorescence microscopy

PRH were seeded into 35 mm culture dishes for 4 h, the medium was replaced. Fresh medium containing 30 MOI Ad-mCherry-GFP-LC3B was added, and the cells were incubated at 37°C for 24 h. After removing the medium containing Ad-mCherry-GFP-LC3B, fresh medium or medium containing different concentrations of MCT (200, 300, and 400 μM) was added, and incubation continued. The distribution of GFP-LC3B green fluorescent puncta and mCherry-LC3B red fluorescent puncta was observed under a fluorescence microscope (OLYMPUS-IX71, Japan).

### 2.6 Apoptosis detection by annexin V/PI double staining

The rate of cell apoptosis was assessed using the Annexin V-FITC Apoptosis Detection Kit (Dojindo, Catalog No.: AD10, Japan) following the manufacturer’s protocol. In summary, PRH were collected and washed twice with cold PBS. The cells were then resuspended in binding buffer and incubated with 5 μL of annexin V-FITC and 5 μL of PI for 15 min at room temperature in the dark. Apoptosis was analyzed using a BD FACSAria™ III flow cytometer (BD, United States) within 1 h, and the data were processed using Treestar FlowJo software (United States).

### 2.7 Statistical analysis

All results are expressed as the mean ± SD (with vertical error bars) from triplicate experiments. Differences between groups were assessed using One-way ANOVA, performed with GraphPad Prism version 10.0 software (San Diego, CA, United States). *p* < 0.05 was indicated a statistically difference and *p* < 0.01 was indicated significantly statistically difference.

## 3 Result

### 3.1 MCT increases the level of autophagic markers in PRHs

**FIGURE 1:**
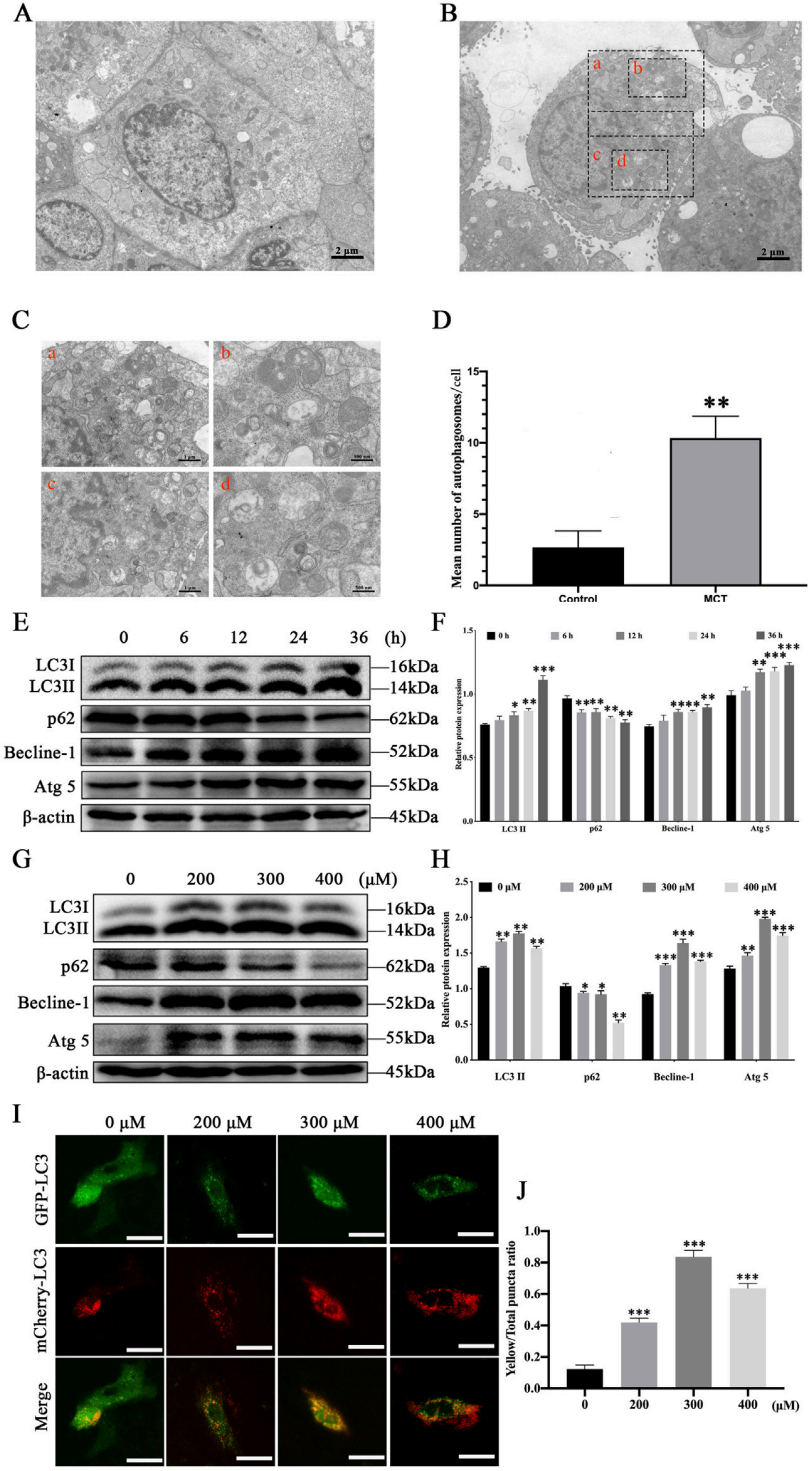
MCT induces autophagy in PRHs. **(A)** The ultrastructure of PRHs not exposed to MCT. **(B)** MCT increases the formation of autophagosome-like vesicles. **(C)** Magnified views of the autophagosome-like vesicles are enclosed by four black square frames **(A–C)** in part **(B)**. Scale bars, 1 μm **(A, C)** and 500 nm **(B, D)**. **(D)** Quantification of the number of autophagosome-like vesicles per cell profile in cells with/without MCT. The average number of the vesicles in each cell was obtained from at least 10 cells per experimental condition. **(E)** Detection of autophagy-related protein expression induced by 300 μM MCT over different time (0, 6, 12, 24 and 36 h), including LC3II, Beclin-1, p62, Atg5 and β-actin (loading control) by Western blot. **(F)** Quantitative analysis of protein levels compared to β-actin protein levels was determined by densitometry in **(E)**. **(G)** Detection autophagy-related protein expression induced by different concentrations (0, 200, 300 and 400 μM) of MCT after 36 h, including LC3II, Beclin-1, p62, Atg5 and β-actin (loading control) by Western blot. **(H)** Quantitative analysis of protein levels compared to β-actin protein levels was determined by densitometry in **(G)**. **(I)** MCT promotes the formation of autophagic fluorescent puncta in PRHs. Scale bars, 100 μm. **(J)** Quantified the yellow fluorescent puncta ratio of autophagy in PRHs. Data are presented as mean ± SD of three independent experiments. *p < 0.05, **p < 0.01, ***p < 0.001 compared to control.

To determine whether MCT induces autophagosome formation, PRHs were treated with 300 μM MCT for 36 h, followed by ultrastructural analysis. Compared to control group (Figure 1A), the number of vesicle-like structures resembling single- or double-membrane autophagosomes significantly increased in MCT-treated cells (Figure 1B). Additionally, regional magnification of the cells revealed that these vesicles contained numerous contents (Figure 1C). In comparison, such vesicle-like structures were rarely observed in control group (Figure 1D). These results indicate that MCT can induce autophagy in PRHs and promote the formation of autophagosome structures.

To further investigate autophagy induced by MCT in PRHs, autophagy-related marker proteins were analyzed using Western blotting. After treatment with 300 μM MCT for 0, 6, 12, 24 and 36 h, the expression levels of autophagy markers LC3II, Beclin-1, and Atg5 in hepatocytes significantly increased over time, while the expression of p62 protein markedly decreased (Figures 1E, F). When cells were treated with different concentrations of MCT (0, 200, 300 and 400 μM) for 36 h, the expression levels of LC3II, Beclin-1, and Atg5 were significantly elevated compared to the control group (Figures 1G, H). Autophagic fluorescent puncta were observed using a fluorescence microscope. As shown in Figures 1I, J, after 36 h of MCT treatment in PRHs, which is infected with the Ad-mCherry-GFP-LC3B, the control group displayed a uniform distribution of GFP-LC3B green fluorescence with only a few bright green fluorescent puncta visible. The mCherry-LC3B red fluorescent puncta were relatively scarce in the cells, resulting in a low number of yellow puncta when they were merged. In contrast, the MCT-treated group showed a significant increase in the number of green fluorescent puncta, along with a marked increase in mCherry-LC3B red fluorescent puncta. Consequently, the proportion of yellow fluorescent puncta in MCT-treated hepatocytes was significantly higher. These results indicate that MCT promotes autophagy in PRHs.

### 3.2 MCT inhibits the expression of the PI3K/AKT/mTOR pathway in PRHs

**FIGURE 2:**
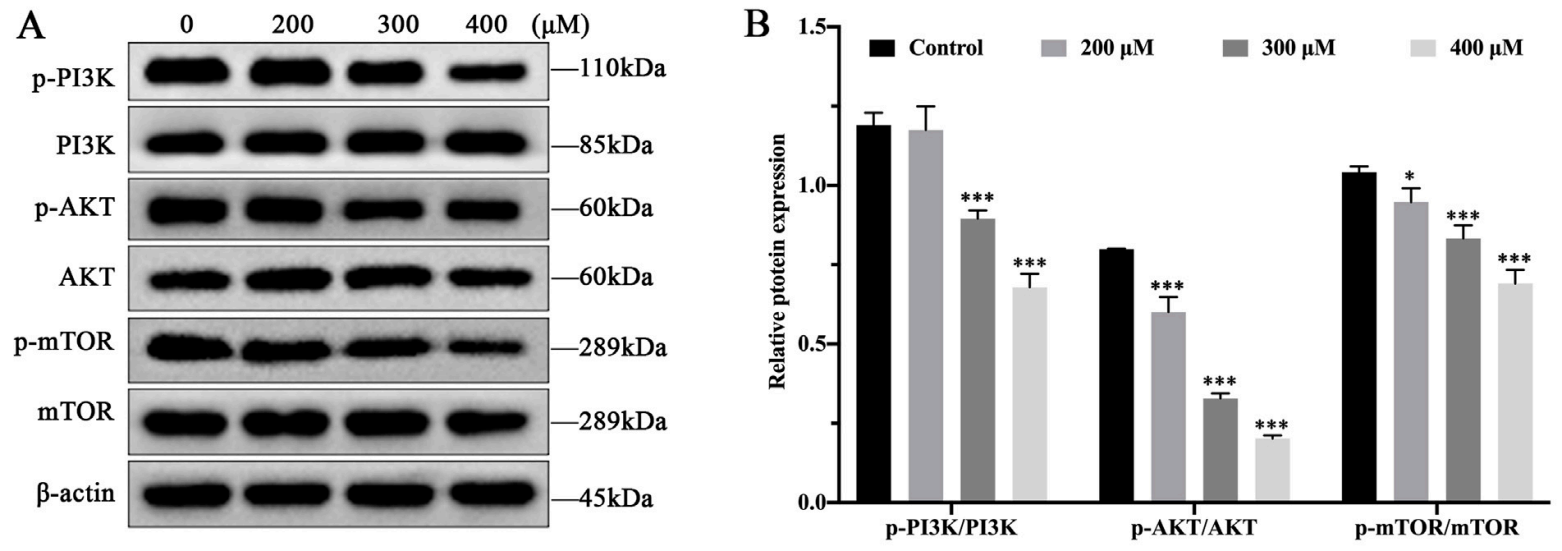
MCT inhibits the phosphorylation levels of PI3K/AKT/mTOR pathway in PRHs. **(A)** Detection autophagy-related protein expression induced by different concentrations (0, 200, 300 and 400 μM) of MCT after 36 h, including PI3K, p-PI3K, AKT, p-AKT, mTOR, p-mTOR and β-actin (loading control) by Western blot. **(B)** The phosphorylation levels of proteins were quantitatively analyzed relative to their corresponding non-phosphorylated proteins using densitometry in **(A)**. Data are presented as mean ± SD of three independent experiments. *p < 0.05, **p < 0.01, ***p < 0.001 compared to control.

To explore the effect of MCT on PI3K/AKT/mTOR pathway, Western blot was used to assess the expression level of p-PI3K, p-AKT, and p-mTOR in PRHs, treated with different concentrations of MCT (0, 200, 300 and 400 μM). As shown in Figure 2, compared to the control group, the expression of p-PI3K, p-AKT and p-mTOR proteins showed a significant reduction with increasing concentrations of MCT. These results suggest that MCT can inhibit the phosphorylation of proteins in the PI3K/AKT/mTOR pathway.

### 3.3 Activation of autophagy enhances MCT-induced apoptosis in PRHs

**FIGURE 3:**
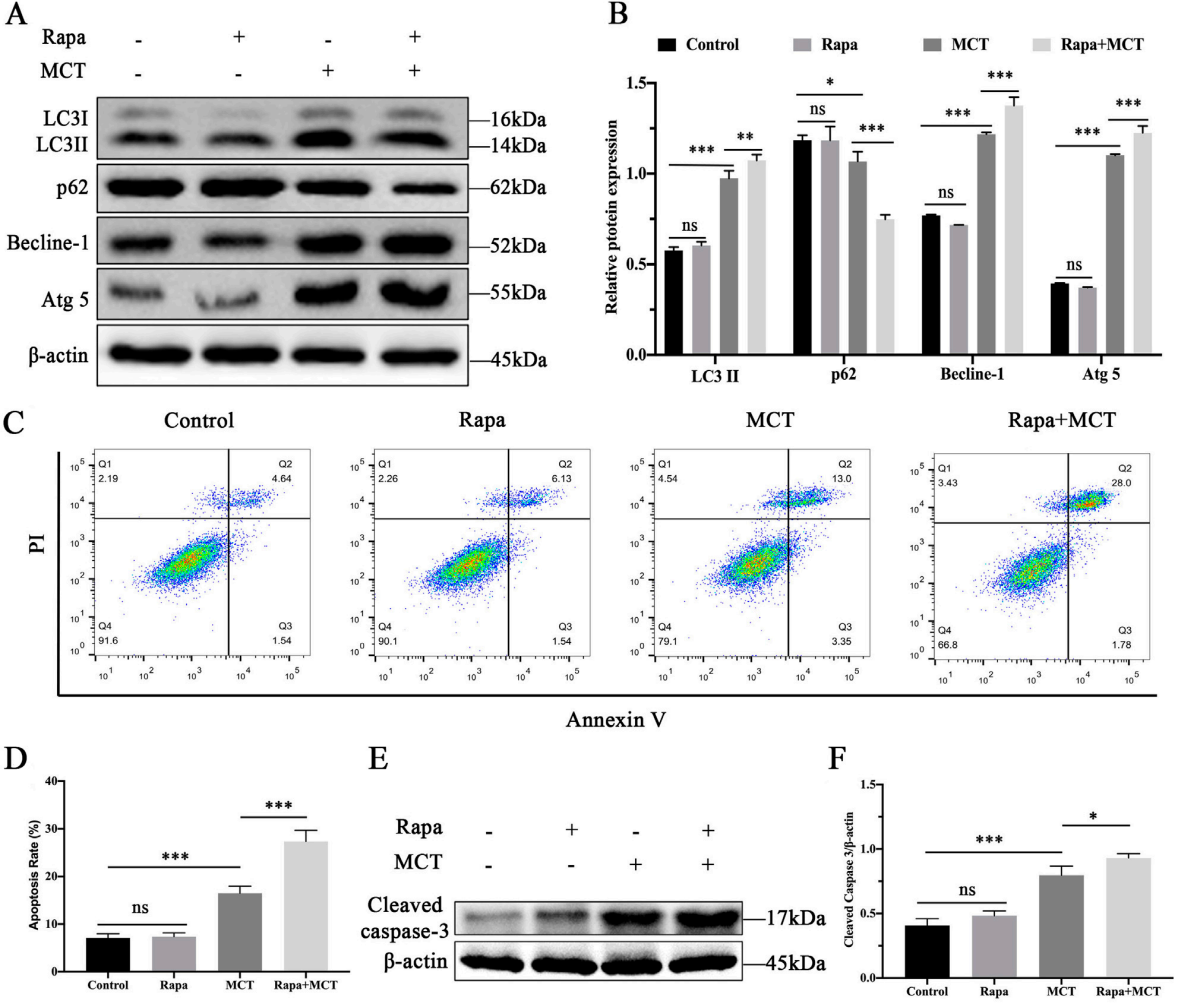
Activation of autophagy enhances MCT-induced apoptosis in PRHs. PRHs were divided into four groups (control, Rapamycin (50 nM)-treated group, MCT (300 μM)-treated group, and Rapamycin (50 nM) + MCT (300 μM)-treated group) for 36 h. **(A)** Detection of autophagy-related protein expression (LC3II, Beclin-1, p62, Atg5 and β-actin (loading control)) by Western blot. **(B)** Quantitative analysis of protein levels compared to β-actin protein levels was determined by densitometry in **(A) (C)** The percentages of apoptosis cells were measured by flow cytometry. Q1 quadrant stands for cell death induced by mechanical damage or necrotic cells, Q2 quadrant stands for late apoptosis cells, Q3 quadrant stands for early apoptosis cells, Q4 quadrant stands for normal cells. The sum of cell apoptosis included the early and late apoptosis cells. **(D)** The results of quantitative analyses of apoptosis rate. **(E)** Detection of cleaved capase-3 by Western blot. **(F)** Quantitative analysis of protein levels compared to β-actin protein levels was determined by densitometry in **(C)**. Data are presented as mean ± SD of three independent experiments. *p < 0.05, **p < 0.01, ***p < 0.001 compared to control.

PRHs were divided into four groups (control, Rapamycin (50 nM)-treated group, MCT (300 μM)-treated group and Rapamycin (50 nM) +MCT (300 μM)-treated group). The expression of autophagy-related proteins was assessed. As shown in Figures 3A, B, compared to the control group, there was no significant change in the expression levels of LC3II, p62, Becline-1, and Atg5 proteins in the Rapamycin-treated group. However, in the Rapamycin + MCT-treated group, the expression of LC3II, Becline-1, and Atg5 proteins was significantly increased, while the expression of p62 was significantly decreased compared to the MCT-treated group. These results indicate that Rapamycin can promote MCT-induced autophagy in PRHs.

We used flow cytometry to confirm the role of Rapamycin in MCT-induced apoptosis in PRHs. As shown in Figures 3C, D, Rapamycin pretreatment did not significantly affect apoptosis. However, the apoptosis rate was significantly increased in the Rapamycin + MCT-treated group. Additionally, Western blot was performed to detect the expression of cleaved caspase-3. As shown in Figures 3E, F, Rapamycin pretreatment did not significantly alter cleaved caspase-3 expression compared to the control group. In contrast, cleaved caspase-3 expression was significantly elevated in the Rapamycin + MCT-treated group compared to the MCT-treated group. These results indicate that Rapamycin can promote MCT-induced apoptosis in PRHs.

### 3.4 Inhibition of autophagy attenuates MCT-induced apoptosis in PRHs

**FIGURE 4:**
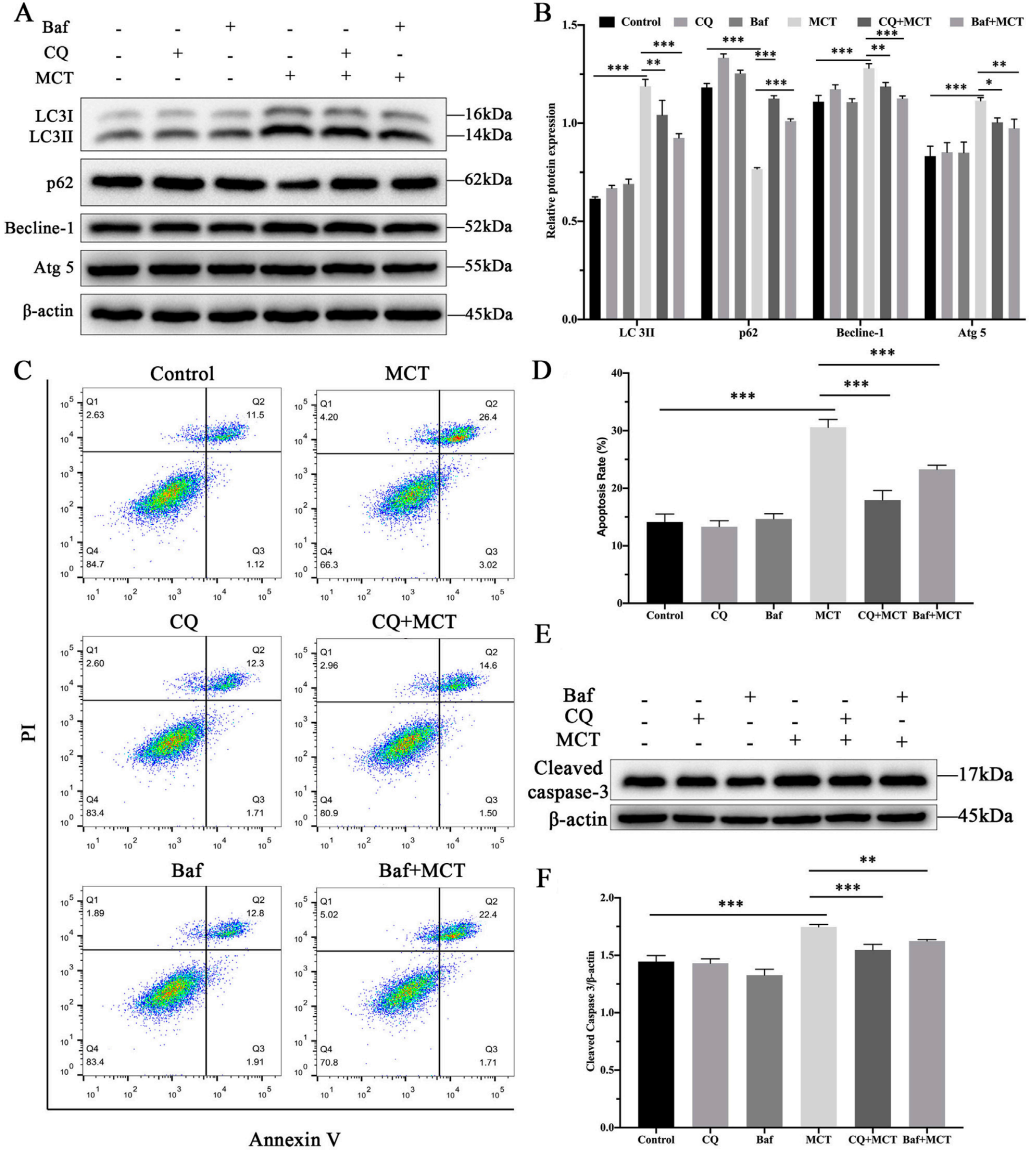
Inhibition of autophagy attenuates MCT-induced apoptosis in PRHs. PRHs were divided into six groups (control, Chloroquine (15 μM)-treated group, Bafilomycin A1 (10 nM)-treated group, MCT (300 μM)-treated group, Chloroquine (15 μM) + MCT (300 μM)-treated group, and Bafilomycin A1 (10 nM) + MCT (300 μM)-treated group) and incubated for 36 h. **(A)** Detection of autophagy-related protein expression (LC3II, Beclin-1, p62, Atg5 and β-actin (loading control)) by Western blot. **(B)** Quantitative analysis of protein levels compared to β-actin protein levels was determined by densitometry in **(A) (C)** The percentages of apoptosis cells were measured by flow cytometry. Q1 quadrant stands for cell death induced by mechanical damage or necrotic cells, Q2 quadrant stands for late apoptosis cells, Q3 quadrant stands for early apoptosis cells, Q4 quadrant stands for normal cells. The sum of cell apoptosis included the early and late apoptosis cells. **(D)** The results of quantitative analyses of apoptosis rate. **(E)** Detection of cleaved capase-3 by Western blot. **(F)** Quantitative analysis of protein levels compared to β-actin protein levels was determined by densitometry in **(C)**. Data are presented as mean ± SD of three independent experiments. *p < 0.05, **p < 0.01, ***p < 0.001 compared to control.

To further investigate the role of autophagy in MCT-induced apoptosis, PRHs were divided into six group (control, Chloroquine (15 μM)-treated group, Bafilomycin A1 (10 nM)-treated group, MCT (300 μM)-treated group, Chloroquine (15 μM) + MCT (300 μM)-treated group and Bafilomycin A1 (10 nM) + MCT (300 μM)-treated group). The expression of autophagy-related proteins was assessed. As illustrated in Figures 4A, B, treatment with MCT significantly upregulated the expression of LC3II, Becline-1, and Atg5, while the levels of p62 were markedly reduced compared to the control group. In contrast, co-treatment with Chloroquine and MCT significantly decreased the expression of LC3II, Becline-1, and Atg5, and increased p62 levels compared to the MCT group. Similarly, Bafilomycin A1 combined with MCT treatment led to a significant reduction in LC3II, Becline-1, and Atg5 expression, accompanied by an increase in p62 levels. These findings suggest that Chloroquine and Bafilomycin A1 effectively inhibit MCT-induced autophagy in PRHs.

Flow cytometry was employed to assess the impact of these autophagy inhibitors on apoptosis. As shown in Figures 4C, D, Chloroquine and Bafilomycin A1 pre-treatment did not significantly affect apoptosis rates compared to the control group. However, when compared to the MCT-treated group, the combination of Chloroquine or Bafilomycin A1 with MCT led to a marked reduction in apoptosis rates. Furthermore, Western blot analysis of the apoptotic marker cleaved caspase-3, as shown in Figures 4E, F, revealed no significant changes in its expression in cells pre-treated with Chloroquine or Bafilomycin A1 alone. In contrast, co-treatment with MCT significantly reduced cleaved caspase-3 levels in the presence of either inhibitor. These findings suggest that inhibition of autophagy attenuates MCT-induced apoptosis in PRHs.

## 4 Discussion

Monocrotaline (MCT), a type of pyrrolizidine alkaloid (PA), is known to cause liver damage. Our previous study has demonstrated that MCT contributes to the development of apoptosis in rat hepatocytes (Guo et al., 2020). Autophagy and apoptosis are interconnected processes involved in PCD, and study has shown that PAs can cause autophagy (Huang et al., 2020). The interaction between autophagy and apoptosis may provide insights into the impact of MCT on hepatocyte function under pathological conditions. Therefore, this study investigates the role of autophagy in MCT-induced apoptosis of PRHs.

Autophagy is fundamental in maintaining cellular energy and metabolic balance. Its role in regulating cell survival varies depending on changes and stimuli in both the intracellular and extracellular environments (Levy et al., 2017). Under certain conditions, basal autophagy was reported to provide the necessary energy and resources for cell survival and effectively prevent chronic damage to tissues and organs, thereby supporting cell viability (Debnath et al., 2023). Conversely, when sustained by environmental stimuli, autophagy can trigger apoptotic pathways, leading to excessive cell death and consequent damage to tissues and organs (Nassour et al., 2019). Our study found that MCT induces autophagy in PRHs, as demonstrated by a marked increase in the number of autophagosomes. Previous research has demonstrated that LC3II expression levels correlate with the number of autophagosomes, while Becline-1 is a key protein involved in autophagosome formation. Atg5 is also a critical component of the autophagosome assembly complex, and the degradation of p62 serves as a marker of completed autophagy (Kan et al., 2022). Our study found a significant increase in LC3II, Becline-1 and Atg5 levels, accompanied by a notable decrease in p62 expression. With fluorescence microscopy, we found that at a concentration of 300 μM MCT, the number of yellow puncta peaked, suggesting enhanced autophagy. However, at a concentration of 400 μM MCT, while the number of yellow puncta decreased, there was a notable increase in red puncta, indicating lysosomal-autophagosome fusion and enhanced autophagic flux. These results indicate that MCT not only promotes the formation of autophagosomes in PRHs but also enhances the level of autophagic flux.

Autophagy is essential for maintaining energy and nutrient balance in the liver and plays a significant role in regulating its pathophysiological processes (Mizushima and Komatsu, 2011). Recent evidence increasingly suggests that the PI3K/AKT/mTOR pathway is a critical regulator of autophagy and is involved in the initiation and progression of various pathological disorders (Yu et al., 2024). In this study, we showed that MCT inhibits the phosphorylation levels of PI3K/AKT/mTOR pathway. It is also one of the primary mechanisms by which MCT regulates autophagy in PRHs.

Numerous studies have established that autophagy is a vital pathway for promoting cell survival under environmental stress, but it can also lead to apoptosis (Nilsson et al., 2013). To elucidate the role of autophagy in MCT-induced apoptosis of PRHs, we examined the alterations in autophagy and apoptosis-associated proteins to better understand the interplay between these two processes. This approach allowed us to gain a more comprehensive understanding of how autophagy influences cell fate under MCT treatment. Rapamycin, a known autophagy activator, facilitates the induction of autophagy through the inhibition of mTOR protein expression (Zhang et al., 2018). Following treatment with the autophagy activator, there was no significant difference observed compared to the control group. However, in the combined treatment with MCT and Rapamycin, a notable increase in the expression levels of LC3II, Becline-1, and Atg5 proteins was observed, along with a significant decrease in p62 protein levels. This suggests an enhancement of autophagic degradation. Furthermore, apoptosis analysis demonstrated that co-treatment with MCT and Rapamycin led to a higher rate of apoptosis in PRHs, as indicated by a significant increase in cleaved caspase-3 protein expression compared to the MCT-treated group. This effect may result from the intensified autophagic flux, which augments cellular degradative processes, disrupts cellular homeostasis, and ultimately triggers cell death.

In this study, Chloroquine and Bafilomycin A1 were utilized as autophagy inhibitors. Chloroquine primarily reduces lysosomal acidity, thereby impairing autophagosome degradation (Deretic, 2021). In contrast, Bafilomycin A1 inhibits the fusion of lysosomes with autophagosomes (Yoshimori et al., 1991). The results showed that the combined treatment of MCT with either Chloroquine or Bafilomycin A1 significantly reduced the expression levels of LC3II, Becline-1, and Atg5, while notably increasing p62 expression compared to the MCT-treated group, indicating a suppression of autophagic degradation. When comparing the apoptosis rates between the MCT-treated group and those treated with a combination of MCT and Chloroquine or MCT and Bafilomycin A1, it was observed that the co-treatment groups exhibited a significant reduction in apoptosis rates, accompanied by a marked decrease in the expression of the apoptosis-related protein cleaved caspase-3. This observation may be due to the blockade of autophagic flux, which mitigates the intense degradation associated with autophagy, thereby maintaining cellular homeostasis and reducing damage to normal cells.

## 5 Conclusion

**FIGURE 5:**
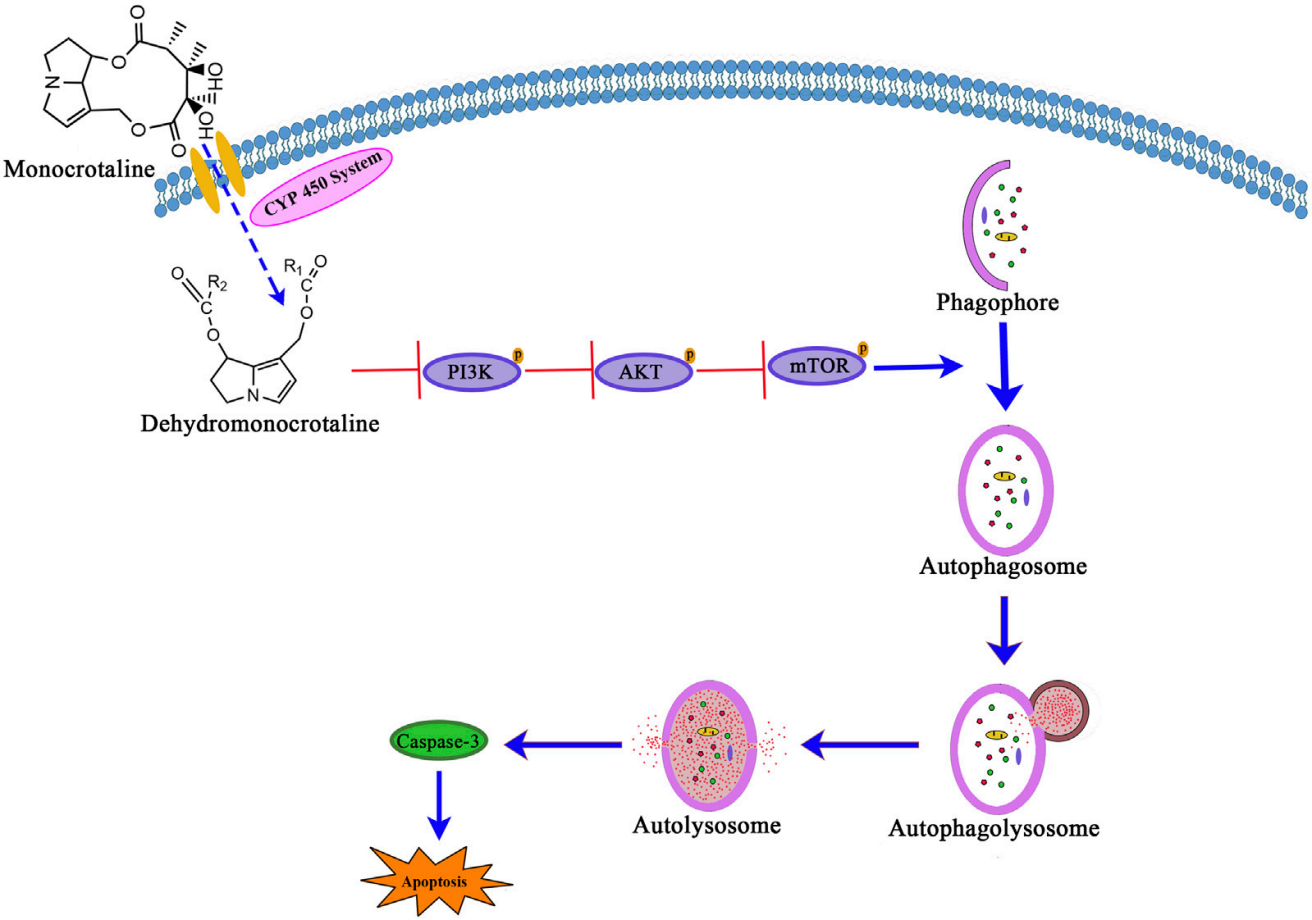
The signaling pathway involved in MCT-induced autophagy via PI3K/AKT/mTOR pathway inhibition leads to apoptosis in PRHs.

In conclusion, our study demonstrates that MCT induces autophagy in PRHs by inhibiting the PI3K/AKT/mTOR signaling pathway (Figure 5). Additionally, autophagy plays a significant role in the hepatotoxicity induced by MCT. Persistent autophagy can lead to excessive cellular metabolism and impair normal hepatic function, leading to PRHs apoptosis.

## Data Availability

The original contributions presented in the study are included in the article/supplementary material, further inquiries can be directed to the corresponding authors.
